# The use of Multispectral Radio-Meter (MSR5) data for wheat crop genotypes identification using machine learning models

**DOI:** 10.1038/s41598-023-46957-5

**Published:** 2023-11-14

**Authors:** Mutiullah Jamil, Hafeezur Rehman, Muhammad Saqlain Zaheer, Aqil Tariq, Rashid Iqbal, Muhammad Usama Hasnain, Asma Majeed, Awais Munir, Ayman El Sabagh, Muhammad Habib ur Rahman, Ahsan Raza, Mohammad Ajmal Ali, Mohamed S. Elshikh

**Affiliations:** 1 Department of Computer Science, Khwaja Fareed University of Engineering and Information Technology, Rahim Yar Khan, 64200 Pakistan; 2 Department of Agricultural Engineering, Khwaja Fareed University of Engineering and Information Technology, Rahim Yar Khan, Pakistan; 3 Department of Wildlife, Fisheries and Aquaculture, Mississippi State University, 775 Stone Boulevard, Mississippi State, MS 39762-9690 USA; 4 State Key Laboratory of Information Engineering in Surveying, Mapping and Remote Sensing (LIESMARS), Wuhan University, Wuhan, 430079 China; 5 Department of Agronomy, Faculty of Agriculture and Environment, The Islamia University of Bahawalpur, Bahawalpur, Pakistan; 6 Institute of Plant Breeding and Biotechnology, MNS-University of Agriculture, Multan, Pakistan; 7 Institute of Agro-Industry & Environment, The Islamia University of Bahawalpur, Bahawalpur, Pakistan; 8 Department of Agronomy, Faculty of Agriculture, Kafrelsheikh University, Kafr El-Shaikh, 33516 Egypt; 9 Department of Field Crops, Faculty of Agriculture, Siirt University, Siirt, Turkey; 10 Crop Science, Institute of Crop Science and Resource Conservation (INRES), University of Bonn, 53115 Bonn, Germany; 11 Leibniz Centre for Agricultural Landscape Research (ZALF), Eberswalder Straße 84, 15374 Müncheberg, Germany; 12 Department of Botany and Microbiology, College of Science, King Saud University, 11451 Riyadh, Saudi Arabia

**Keywords:** Plant sciences, Environmental sciences

## Abstract

Satellite remote sensing is widely being used by the researchers and geospatial scientists due to its free data access for land observation and agricultural activities monitoring. The world is suffering from food shortages due to the dramatic increase in population and climate change. Various crop genotypes can survive in harsh climatic conditions and give more production with less disease infection. Remote sensing can play an essential role in crop genotype identification using computer vision. In many studies, different objects, crops, and land cover classification is done successfully, while crop genotypes classification is still a gray area. Despite the importance of genotype identification for production planning, a significant method has yet to be developed to detect the genotypes varieties of crop yield using multispectral radiometer data. In this study, three genotypes of wheat crop (Aas-‘2011’, ‘Miraj-‘08’, and ‘Punjnad-1) fields are prepared for the investigation of multispectral radio meter band properties. Temporal data (every 15 days from the height of 10 feet covering 5 feet in the circle in one scan) is collected using an efficient multispectral Radio Meter (MSR5 five bands). Two hundred yield samples of each wheat genotype are acquired and manually labeled accordingly for the training of supervised machine learning models. To find the strength of features (five bands), Principle Component Analysis (PCA), Linear Discriminant Analysis (LDA), and Nonlinear Discernment Analysis (NDA) are performed besides the machine learning models of the Extra Tree Classifier (ETC), Random Forest (RF), Support Vector Machine (SVM), Decision Tree (DT), Logistic Regression (LR), k Nearest Neighbor (KNN) and Artificial Neural Network (ANN) with detailed of configuration settings. ANN and random forest algorithm have achieved approximately maximum accuracy of 97% and 96% on the test dataset. It is recommended that digital policymakers from the agriculture department can use ANN and RF to identify the different genotypes at farmer's fields and research centers. These findings can be used for precision identification and management of the crop specific genotypes for optimized resource use efficiency.

## Introduction

Timely and precise crop yield estimation is the pre-request to define the food availability of a nation. In the modern world, every agricultural country can acknowledge the importance of precise knowledge of crop conditions for plant management. It is the most critical single economic sector, contributing 24% of the national income of Pakistan. Its significance prevails as 48% of the working population is engaged in this sector. Punjab is the country’s primary producer of cash crops^1–3^. In such circumstances, improving agricultural land monitoring is among the most critical and pressing prominent issues that Pakistan must address. Crop patterns have their importance as they are used to develop regional strategies and programs to increase farm production and efficient use of land resources^4–6^.

One agricultural production goal is maximizing crop yield while minimizing costs. Early detection and management of seasonal crop yield indicator problems can help to increase production and subsequent profit. Crop yield spatial variability can be assessed using remote sensing and global positioning systems (GPS)^7–13^. Recently, crop yield prediction has been observed in various literature before harvest^14,15^. Crop yield spectral characteristics without introducing weather noise. A Multispectral Radio Meter with five bands (MSR5) is used for land use land cover (LULC) using K Nearest Neighbor (KNN)^14,16,17^.

Various types of crops such as sugar cane, potato, tobacco, and land change detection can be made using remotely sensed data such as MSR5, Unmanned Aerial Vehicle (UAV), Landsat 8, and Sentinel. The use of multispectral radiometers in agriculture is becoming a novel approach to identifying insects, pests, and genotypes as well. It is a digital sensor optics with high microelectronics that provides a spectral record of the light to identify the objective.

Although, satellite or Aerial Remote Sensing (ARS) technology can significantly improve the present systems to acquire and generate agricultural resource data^18–20^. In most of the literature, researchers focus on the identification of different classes (Bare land, water, sugarcane, tomato, potato, etc.) of LULC classification. Another issue in developing countries such as Pakistan, Afghanistan, and India tried to cultivate regional trended crop yield genotypes of wheat and rice, the information of trending crops is gathered through a Decision Tree Classification (DTC) approach that is costly, time-consuming, and with a high error rate but it is not being considered with machine learning methods^21–23^. Pakistani wheat yield has increased (more than 1%)^24^. New varieties and genotypes have had an important role in improving crop production in recent decades^25^. Now it is necessary to adopt new management technologies and policies to adopt the new varieties.

Genotype crop classifications are still ignored while varieties of various crops have a high impact on increasing the production of crop yield^5,6,17,26–30^. The DTC and RF^14,31^, models involve various parameters: weather conditions, water stress, rainfall, air humidity, and temperature^7,32,33^. Wheat varieties are an important parameterthat is absent from the Artificial Neural network (ANN) and KNN models due to the unavailability of an identification method. That is the reason; this work mainly focuses on the robust machine-learning model for the identification of wheat genotype and variety.

Multispectral radiometers capture the electromagnetic radiation reflected or emitted by objects in different spectral bands, allowing for the collection of valuable crop-related information. These sensors can capture various spectral bands, ranging from visible to near-infrared and thermal infrared, providing a wealth of data about crop health, vigor, and other important characteristics. Machine learning models, such as support vector machines (SVMs), random forests, and artificial neural networks, have demonstrated great potential in analyzing and interpreting complex data patterns^34–36^. By training these models on multispectral radiometer data, it becomes possible to develop robust and accurate classification models for identifying different wheat crop genotypes. Number of researchers use different machine learning methods used for classification puposes.

Integrating machine learning models Support Vector Machine (SVM)^37^, Extra Tree Classifier (ETC)^38^, Logistic Regression (LR)^39^, KNN^40^ and Decision Tree (DT)^14^ with multispectral radiometer data can potentially revolutionize wheat crop management practices. Accurate and rapid genotype identification can significantly aid plant breeders and agronomist in selecting superior genotypes with desired traits, improving crop yield and quality. Moreover, this approach can contribute to precision agriculture by enabling targeted interventions, such as optimized fertilizer application, pest management, and irrigation strategies.

The application of machine learning models across several domains has extended to the agricultural sector, resulting in notable advantages for this industry. For example, Fei proposed an ensemble framework for wheat yield prediction with different water treatments^41^. The authors developed Elastic Net Regression (ELR) for the prediction and deployed it with selected features. The proposed ELR achieved 0.729 R^2^ scores by combining the predicted values of all growth stages. This study proposed a machine learning approach for wheat yield prediction using advanced sensing techniques^16,25,42^. The authors proposed XY-fused Networks (XY-Fs), supervised Kohen networks, and counter-propagation artificial neural networks (CP-ANNs) models for this purpose and achieved 81.65% accuracy using supervised Kohen networks.

The machine learning model, the satellite, and climatic data are integrated to predict the wheat crop yield^43^. The source data taken from 2000 to 2014 from Australia is used at the statistical division level. They deployed LASSO, RF, neural networks, and SVM models for the prediction of a significant accuracy score of 0.75 R^2^. The winter wheat yield prediction using machine learning models from multi-source data in China^41^. They combined climate, soil, and remote sensing data to predict winter wheat yield based on the Google Earth Engine. The achieved R^2^ > 0.75 with SVM, RF, and Gaussian process regression.

In machine learning models, the solar-induced chlorophyll fluorescence data is used to predict the wheat yield^44^. They deployed LASSO, extreme gradient boosting, Support Vector Regression (SVR), ridge regression, RF regression, and Long Short Term Memory (LSTM). SVR outperforms all other models as well as the deep learning models LSTM with a significant R^2^ of 0.87. UAV hyperspectral and ensemble machine-learning approaches to predict the wheat yield^45^. Three techniques of feature selection such as Brute feature selection, recursive feature elimination, and the Pearson correlation coefficient. They combined four machine-learning models to make an ensemble: SVM, RF, Gaussian process, and linear ridge regression. The ensemble model achieved a 78% accuracy score.

Environmental and phenological data can predict winter wheat yield using convolutional neural networks^46^. They collect data from 271 counties in Germany and deploy several machine learning and deep learning models. The proposed model convolutional neural networks achieved 7–14% lower RMSE and 3–15% lower MAE. An approach for wheat yield prediction using kernel ridge regression and Satellite-derived predictors^47^. They combined kernel ridge regression, complete ensemble empirical mode decomposition with adaptive noise (CEEMDAN), and the grey wolf optimizer (GWO-CEEMDAN-KRR). Compared to baseline models, the proposed model reduces the error rate by 20%. Similarly, an approach for regional and local-scale wheat yield prediction using RF in Australia^48^. RF achieved a significant 0.89 R^2^ score for Victoria region data.

Multispectral images were collected from a UAV platform to monitor maize growth and nutritional status^49^. The researchers apply radiometric calibration and establish linear regression relationships between SPAD values and spectral/textural indices. Machine learning models, specifically support vector machine (SVM) and random forest (RF), are employed to estimate SPAD values, with SVM performing better (R^2^ = 0.81, RMSE = 0.14). A comprehensive review of the application of machine learning in agricultural production systems^50^. The review covers various areas such as crop management, livestock management, water management, and soil management. Machine learning techniques include yield prediction, disease detection, weed detection, crop quality assessment, species recognition, and management systems that offer valuable insights and recommendations for informed decision-making by farmers. It is hard for humans to estimate and analyze the crop condition to take the necessary action to save resources with maximum output. In the current era, satellite communication costs have become cheap, and it is the best way to monitor objects and earth situations with increased efficiency and precision.

This research study aims to fill the existing gap in the literature by investigating the impact of multispectral radiometer data on wheat crop genotype identification using state-of-the-art machine learning models. The study will evaluate the performance of different machine learning algorithms, assess the effectiveness of feature extraction techniques, and analyze the influence of varying environmental conditions on classification accuracy. The outcomes of this research can have significant implications for wheat breeding programs, precision agriculture, and crop management practices. By harnessing the power of multispectral radiometer data and machine learning models, accurate and efficient genotype identification can contribute to sustainable agriculture, food security, and the optimization of wheat crop production.

## Materials and methods

### Study area

**Figure 1:**
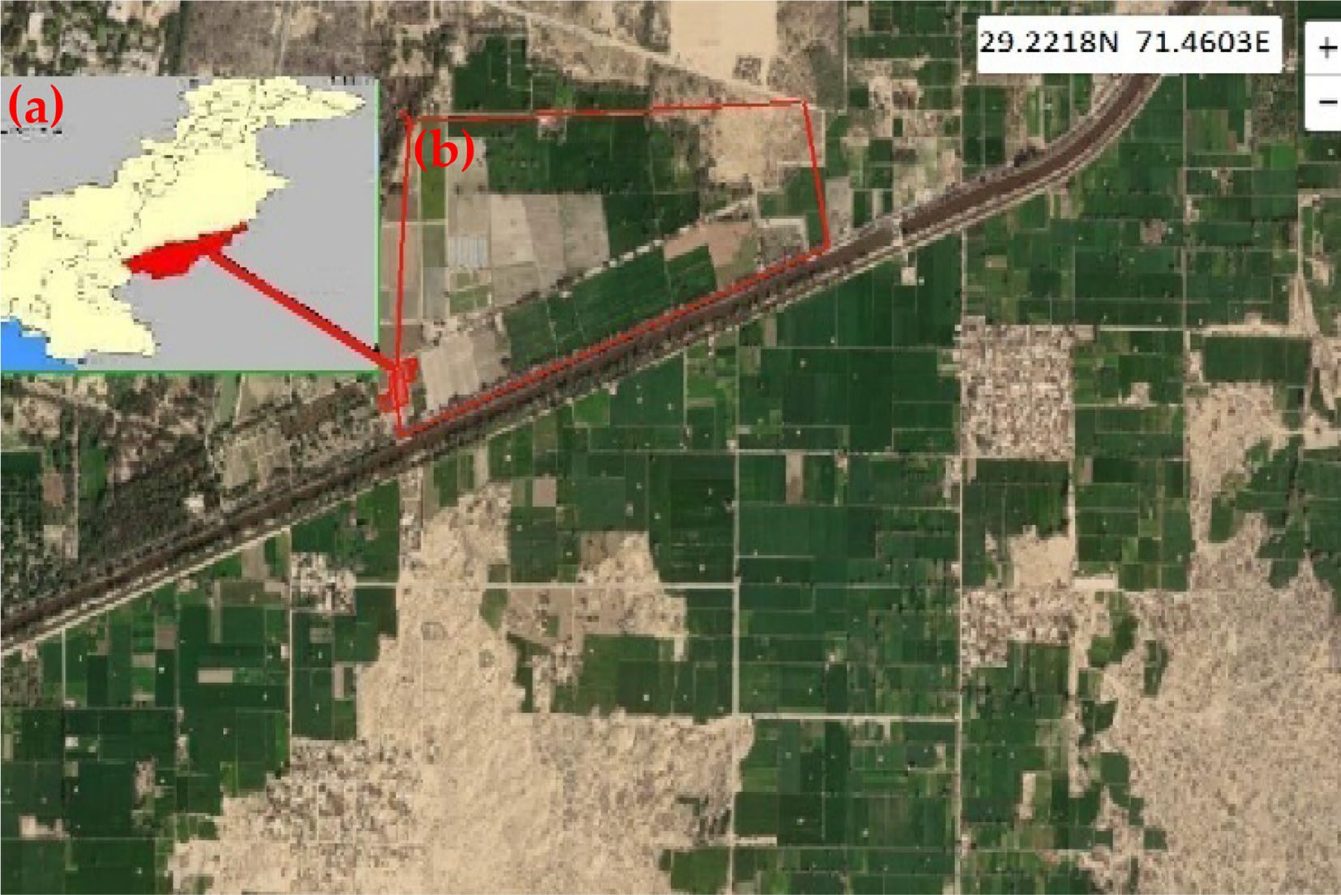
(**a**) Location of the study site using Google earth view with the map of Pakistan and (**b**) image highlighted in red color ROI at the upper top left corner of the image. Google Earth 6.0, (2022).

The study area is the agricultural research center under the Islamia University of Bahawalpur in Bahawalpur City, Punjab, Pakistan, as shown in Fig. 1. For the present study, the site is located at latitude 29°22′18′′ N and longitude 71°46′03′′ E in the agriculture forms of The Islamia University of Bahawalpur^51–54^. The temperature of Bahawalpur is extremely high, and it faces a water stress problem in most of the regions^55^. The study region is very diverse and incorporates Punjab agro-climates with a minimum rainfall range of 2 mm/month in the driest month. October is the driest month, and July is the wettest month, with rainfall of 61 mm. Extremely high temperatures and rain intensity cause much of the rainfall evaporation and runoff^56^. In dry areas, water stress plays an important role in decreasing the production of wheat crops.

### Data acquisition and preprocessing

#### Mutispectral radiometric datasets

Satellite remote sensing technology is the modern technology in the development of RS technology, but it has large information with a low resolution of the image. UAVs have low-resolution images compared to satellite images, which cover a large area with special resolution. To accomplish the goal, we need to investigate the power of spectral bands to categorize the genotypes of the wheat crop. Since, we are growing a small amount of wheat for this experiment, using a handheld device is the most efficient way to collect the necessary data^57^. To acquire multispectral radiometric data, the three wheat varieties (genotypes) that were approved by the Punjab seed certification department, Punjab named ‘Aas-‘2011’, ‘Miraj-‘08’, and ‘Punjnad-‘1’ were harvested in three different adjacent plots. Seed grains were provided by the Agricultural Research Department of the Islamia University of Bahawalpur, and each type of wheat variety was kept under the observation of an agricultural research expert. In this way, three plots were built, Ass-2011, Miraj-08, and Punjnand-1, in a row (1 × 3) plot of equal size of 225 square feet each. Additionally, it is ensured that the same human expert does all the harvested processes to reduce the other cropping factors like water, preparation of land, and nutrition supply to the crops.

Various researchers used multispectral radiometers for recording the incoming radiation and light reflectance from the canopy in five spectral bands, similar to Landsat 8 (OLI/TIRS) and Landsat 7 (ETM +) satellites^58,59^. The output data consists of five bands, detail of which is given in Table 1. Each band has a half-peak band of approximately 5–15 nm, depending on the specific band. In this way, MSR5 describes a complete scene based on five numeric digits, i.e., five energy bands. Previous research shows that only a combination of five bands can classify a complete captured scene. This device has already been used for crop classification^54^ and to efficiently measure nitrogen contents and biomass in plants^60^. Table 1 shows the wavelength and spatial resolution for the wheat crop scan used in this study. To assess the crop field data attained at six stages using crop scan MSR5 (for radiometric data) was acquired from different regions of the crop field.

**Table 1:** Wavelength and spatial resolution of the crop scan MSR5 were used in this study.

Spectral band	Wavelength (nm)	Spatial resolution in radios (meter)
Band 1 Blue	450–520 nm	1.524
Band 2 Green	520–630 nm	1.524
Band 3 Red	630–690 nm	1.524
Band 4 SNIR	760–900 nm	1.524
Band 5 FNIR	1550–1750 nm	1.524

Six hundred (600) scans from three fields of the crops as mentioned above i.e., three wheat varieties (Ass-2011, Miraj-08, and Punjnand-1), have been acquired at 10 feet from the ground level. The scanned data was stored in the memory of the Data Logger Controller (DLC) device. It was then transferred to a CSV file to analyze the data by using the routines provided by the vendor of MSR5.

#### Field sample data

The research utilized GPS field surveys and Google Earth images as reference points. It was determined that there are three distinct types of wheat. Visual interpretation of field validation and images from Google Earth were used to select the samples. After that, the ground sample points were arbitrarily divided into sections (80% training and 20%), and the accuracy was computed. The 240 samples out of 300 of each wheat crop variety are taken for training for the possibility of inter-classification of wheat crops using MSR5 data. At the same time, 20% of the whole dataset is randomly selected as test data. RF, SVM, and customized ANN have been selected for classification.

### Crop classification method

#### Features described for crop classification

Previous research demonstrated that the utilization of spectral information to derive the mean, standard deviation, and variation of each band can differentiate between the many characteristics that are associated with crop varieties^16,61–65^. This information is related to the structure of the target surface and the surrounding environment, which can also indicate spatial variation in land cover. So, statistical, structural, and spectral methods can be used to pull out the information about the texture. Previous research has shown that using spectral data to figure out each band’s mean, standard deviation, and variation is a good way to find the difference between the many characteristics of different crop varieties^66^. Significant evidence suggests that the identification of crops can benefit significantly from the use of textural characteristics derived from satellite images^66,67^. The information about the crop’s texture depicts the crop’s density as well as its shape. The spectral information of red-edge bands in the MSR52 data demonstrates a possible performance use in determining the growing state of crops.

#### Classification and assessment accuracy

The retrieved characteristics were used in conjunction with three different advanced machine learning and classification approaches, namely SVM, ANN, and RF. The SVM technique seeks to determine the ideal hyperplane in the n-dimensional space used for classification in order to maximize the margin of separation between classes (the crops)^68–70^. We employed the SVM classifier by making use of LIBSVM and a radial basis function (RBF) kernel^68^. ANN is able to imitate the recognition structure of the human brain and nervous system while maintaining a high degree of non-high linear classification ability^31,71^. One sort of neural network that sees widespread use is known as the multi-layered perceptron. This particular variety of ANN typically consists of three or more layers that can partition nonlinear data^72,73^. It is usual practice to represent the RF classifier as an ensemble of decision trees, with voting serving as the mechanism for assigning class labels. It is capable of dealing with high-dimensional data and is resistant to overfitting to a certain extent^74^. RF is also used to assess the relevance of characteristics in the classification process. These features include texture, spectral, and indices features^14,75–78^.

**Figure 2:**
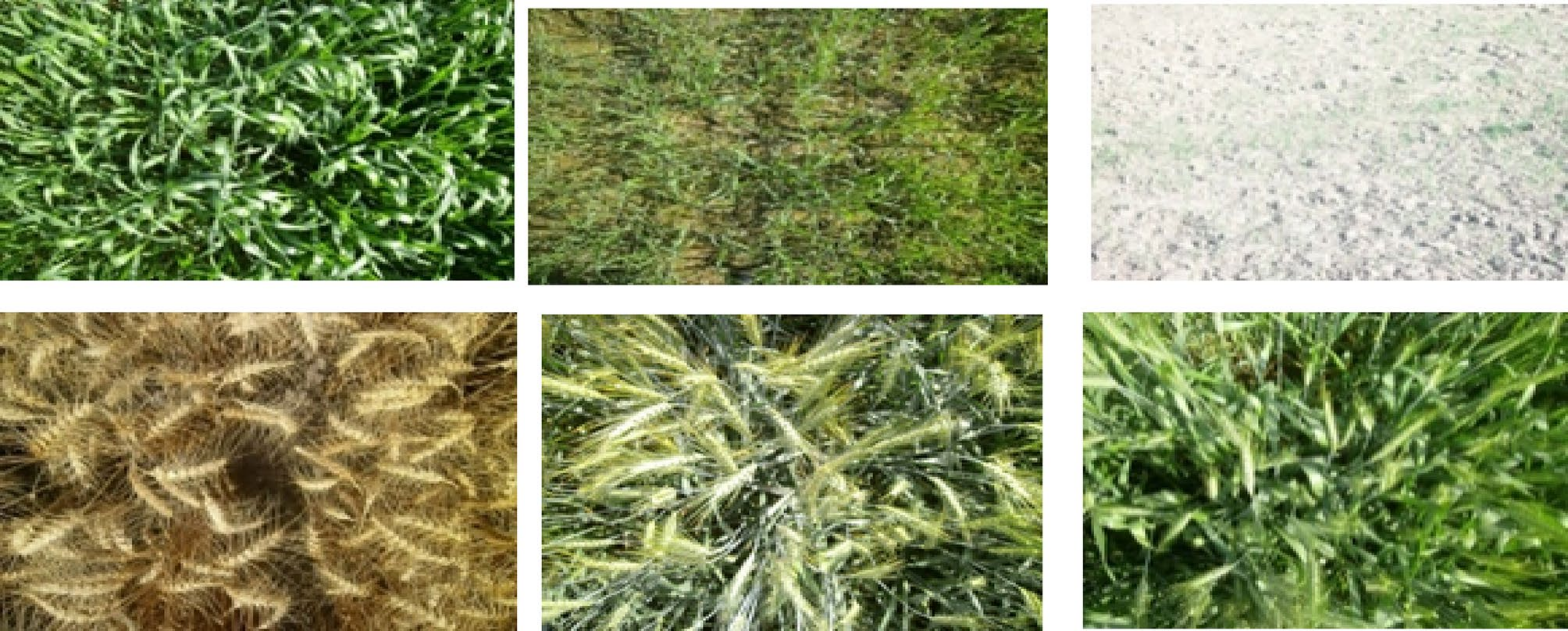
Photographic representation of Wheat Crop of six stages of MSR5 scan data stage 1–3 left to right in the first row and 4–6 in the second row.

This study uses CROPSCAN DATA Inc. 2018 MSR5 multispectral radiometer sample data to train the machine learning model RF, SVM, and ANN with various settings for three different types of wheat crops. A photographic representation of each stage is given in Fig. 2.

**Figure 3:**
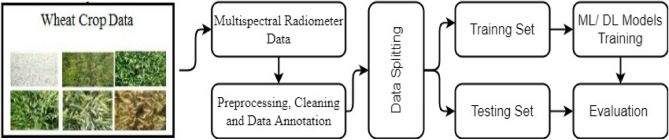
The method used to classify the genotype wheat varieties.

Five columns of Table 2, namely “B”, “G”, “R”, “NIR”, and “SIR” represent reflectance bands of the cropped image. The last column of Table 2 represents the labels of three varieties. Figure 3 shows the methodology adopted for wheat crop classification. The collected data is preprocessed, cleaned, and annotated manually for machine learning models.

**Table 2:** Sample data of multispectral data.

Sr. No.	B	G	R	NIR	SIR	Class
1	2.38	4.23	2.66	48.29	13.28	Aas-2011
2	2.06	4.3	2.51	47.95	13.36	Miraj-08
3	2.57	4.19	3.23	39.4	12.29	Punjnad-1

According to the literature of the last five years published on remote sensing, satellite images of Landsat 7, 8, and Sentinel 2 datasets are trained by RF, SVM, and ANN predominantly, so this study selects these models^14,31,48,51,79^. Most of the researchers prefer to use RF and SVM instead of deep learning models. Usually, a deep learning model is used to enhance the accuracy of classification or mapping of high-resolution images or NDVI image segmentation^80,81^. In this research, the primary focus is to find the strength of five bands of MSR5 for the inter-classification of wheat crop verities so that popular machine learning models are implemented as mentioned above.

We used Python statistical software and the Scikit-learn package to implement these classification techniques^76^. Further, thirteen feature scenarios were tested with the machine learning methods. The classification accuracy is reported for each scenario, and the classification results were compared based on accuracy in crop mapping for each crop class, as described in section “Classification results and accuracy assessment by ML models”. Finally, we calculated a confusion matrix for each classification result based on the ground control points. Then, the overall accuracy (OA), Kappa coefficient, producer’s accuracy (*PA*), and user’s accuracy (*UA*) were calculated to evaluate the classification results^76,82^. Another commonly used performance evaluators are accuracy, Precision, and recall, in which accuracy indicates how many of the total predictions were correct. Precision, also known as positive predictive value, tells how many positively predicted instances were actually true.

In contrast, recall, also known as sensitivity or true positive rate, measures how many of the actual positive instances were correctly predicted as positive. Mathematical formulas are given in Eqs. (1,2 and 3), respectively.

1 $$ Accuracy = \frac{{{\text{True Positives}} + {\text{True Negatives}}}}{{\text{Total Population}}} \times 100\% $$

2 $$ {\text{Precision}} = \frac{{\text{True Positives}}}{{{\text{True Positives}} + {\text{False Positives}}}} \times 100\% $$

3 $$ {\text{Recall}} = \frac{{\text{True Positives}}}{{{\text{True Positives}} + {\text{False Negatives}}}} \times 100\% $$

The *F1* measure (Eq. 4) was calculated to evaluate the effectiveness of the crop classification^83–88^. The *F1* and overall accuracy are considered more meaningful than the Kappa coefficients. The value range of *F1* is from *0* to 1—the larger the *F1* score is, the more accurate the classification results are. The F1 score is the harmonic mean of U and P as shown in (Eq. 4):

4 $$ {\text{F}}1 = 2 \times \frac{{{\text{P}} \times {\text{U}}}}{{{\text{U}} + {\text{P}}}}{ } $$

An additional parameter for image classification accuracy is the Figure of Merit (FoM)^89,90^. The FoM computes from omission, commission, and overall agreement (Eq. 5):

5 $$ FoM = \frac{{\upalpha }}{{{\text{o}} + {\upalpha } + {\text{c}}}} \times 100\% $$

In the Eq. (2), $\mathrm{\alpha }$ represents overall agreement, $\mathrm{o}$ represents overall omission numbers, $\mathrm{c}$ represents overall commission numbers.

### Plant guidelines

All the plant experiments were in compliance with relevant institutional, national, and international guidelines and legislations.

## Results

### Dimensionality reduction techniques with graphical representation of data clusters

**Figure 4:**
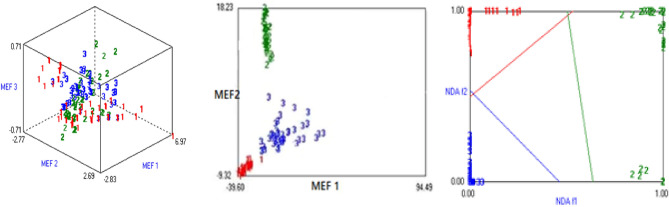
A graphical representation of PCA, LDA, and NDA left to right with Most Expressive Feature (MEF 1, 2 and 3) where 1 = Aas-2011, 2 = Miraj-8 and 3 = Punjnad-1.

Principal component analysis (PCA), linear discriminant analysis (LDA), and nonlinear discriminant analysis (NDA) are popular feature reduction techniques with maximum classification accuracy. It can map the input data from the original space to the new feature space so that all classes are duly clustered and well separated using top-ranked minimum features. These are implemented with MSR5 data, which is normalized by dividing the maximum value found in the data. Its graphical representation is given in Fig. 4, and obtained 93%, 94%, and 94% classification accuracy PCA, LDA, and NDA respectively. It means we can train the ML model and achieve more than 94% accuracy, as shown in Table 7.

### Classification results and accuracy assessment by ML models

Several researchers published their work in remote sensing and LULC classification using RF, SVM, and ANN machine-learning models for extra classification. Therefore, in the intra-classification of wheat crop varieties classification, we implemented the ANN back propagation machine-learning model and did an empirical analysis of the various configuration of ANN, like the number of iterations, learning rate, and several hidden layers. Detailed experiment results of configuration, training, and testing accuracy percentage are given in Table 3.

**Table 3:** Training and testing accuracy of ANN at various configurations.

S. No.	1st hidden layer	2nd hidden layer	(η)	Iterations	Training accuracy	Testing accuracy
1	1	2	0.05	50	32.50	33.33
2	1	2	0.15	50	64.20	65.55
3	1	2	0.25	50	32.40	33.33
4	1	2	0.35	50	65.50	66.67
5	1	2	0.45	50	57.77	58.33
6	1	2	0.55	50	63.75	76.77
7	1	2	0.65	50	63.45	65.00
8	1	2	0.75	50	63.45	58.33
9	1	2	0.85	50	57.44	61.33
10	1	2	0.99	50	56.70	58.33
11	2	2	0.05	100	94.60	90.00
12	2	2	0.15	100	95.50	90.00
13	2	2	0.25	100	93.40	89.33
14	2	2	0.45	100	95.90	90.00
15	3	2	0.05	100	96.40	90.00
16	3	2	0.15	100	96.40	84.00
17	3	2	0.45	100	95.00	80.00
18	4	2	0.15	100	97.90	85.00
19	4	2	0.25	100	97.91	96.67
20	4	2	0.35	100	97.92	45.00
21	4	2	0.35	300	95.80	51.77
22	4	2	0.35	50	96.70	70.00
23	5	2	0.15	100	97.92	85.00

**Figure 5:**
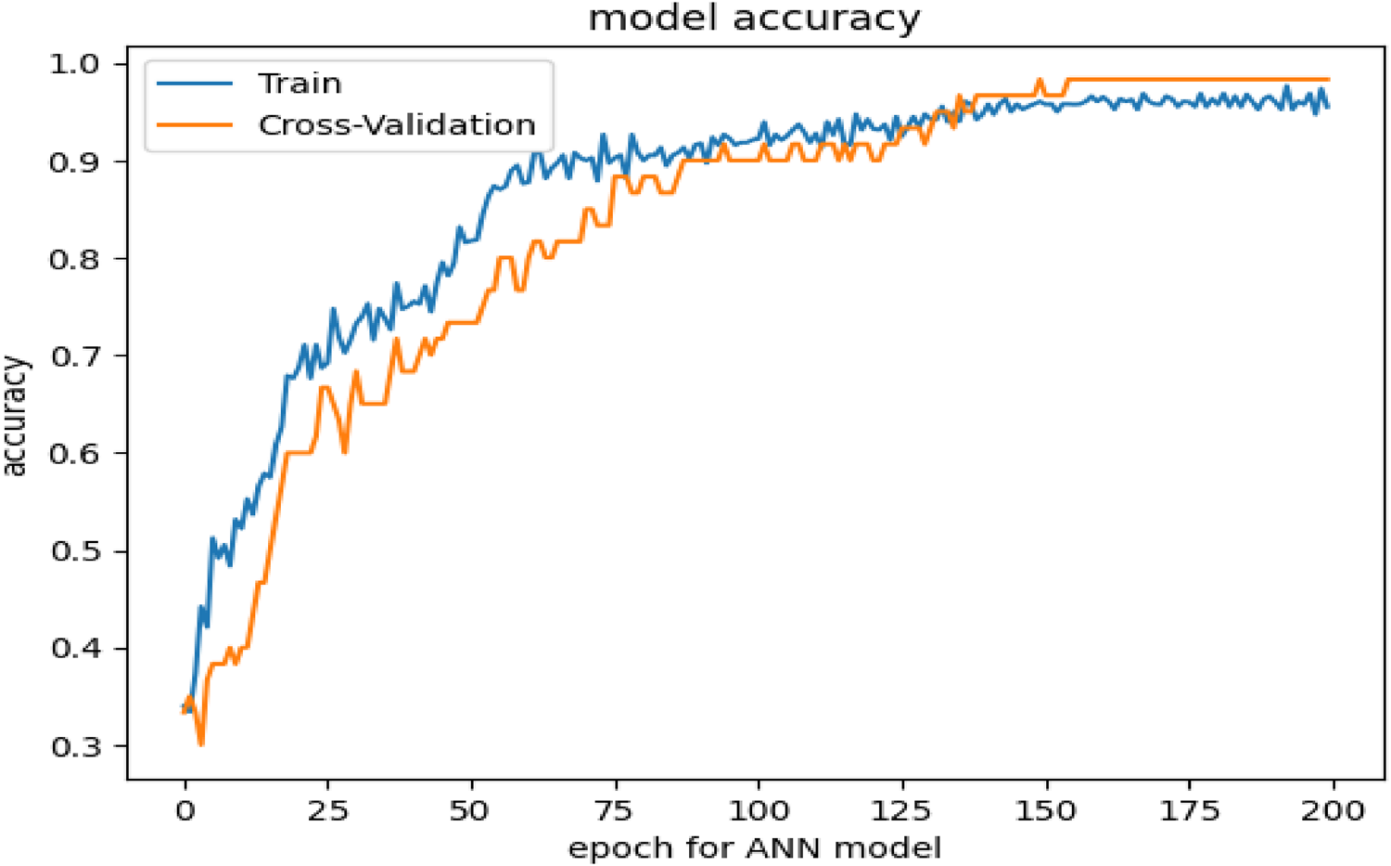
Accuracy score of ANN Machine learning models.

Table 3 shows that the learning rate (η) can play an important role in getting the maximum local value of accuracy, which can achieve a very small change of (η) from 0.01 to 0.15. There is no need to jump from 0.05 to 0.99 maximum because the algorithm is very sensitive to small changes. On the other hand, it is observed that after 0.01 to 0.20 outcome of the algorithm is repeated rather than improved in terms of training and testing accuracy. A number of the first hidden layers are impotent to enhance training and testing accuracy. It is analyzed that when the number of the first hidden layer is increased, the algorithm gives its maximum performance in terms of accuracy with an (η) rate of 0.05 or 0.15 or a maximum at 0.25. When we reached the PCA accuracy, no improvement was found due to the increased number of the hidden layer. Maximum training accuracy is obtained in rows number 20 and 23 in Table 3, also which can be observed in Fig. 5 but the model is over-trained because testing accuracy moves down from 96 to 85%. It means that the model is leading to overfitting.

Neural Network back propagation gives maximum accuracy of 97% and 96% in training and testing datasets, respectively, with a learning rate (**η)** of 0.25. The confusion matrix of the training and testing data set is given in Table 4, the result of the random fores, and the random forest result in Table 5.

**Table 4:** Confusion matrix of ANN with training and testing dataset.

Phase	Class	Aas-2011	Miraj-08	Punjnad-1
Training	Aas-2011	80	0	0
	Miraj-08	0	80	0
	Punjnad-1	5	0	75
Testing	Aas-2011	20	0	0
	Miraj-08	0	20	0
	Punjnad-1	1	1	18

**Table 5:** The result of the Random Forest decision tree model on train dataset.

Class	Aas-2011	Miraj-08	Punjnad-1
Aas-2011	76	0	4
Miraj-08	0	80	0
Punjnad-1	3	1	76
Correctly classified instances		232	97.0533%
Incorrectly classified instances		8	2.9467%
Kappa statistic		0.8972	
MSE		0.1168	
RMSE		0.169	
Total number of instances		240	

### Comparisons of results with models evaluation

To check the model’s performance, we implemented another well-known machine-learning model also used in previous research^91^. Compared the machine learning models’ performance with the proposed ANN model to show the significance of ANN^92^. We used ETC, RF, SVM, DT, LR, and KNN. We deploy these models with their best hyper-parameters settings. RF, ETC are used with 300 estimators indicating that 300 decision trees will be used for weak learners, and each tree will grow to a maxim of 10 level depth because we used ‘max_depth’ parameters with a value of 10. DT is used with only the ‘max_depth’ parameter, which will restrict each model to grow up to a maximum 10-level depth to reduce complexity and overfitting. SVM is used with linear kernel, and LR is used with saga solver. Hyper-parameters for all machine learning models are provided in Table 6.

**Table 6:** Hyper-parameters are used for machine learning models.

Model	Hyper-parameters
ETC	n_estimators = 300, max_depth = 10
RF	n_estimators = 300, max_depth = 10
SVM	Kernel = linear, C = 1.0
DT	max_depth = 10
LR	Solver = saga, C = 1.0

**Figure 6:**
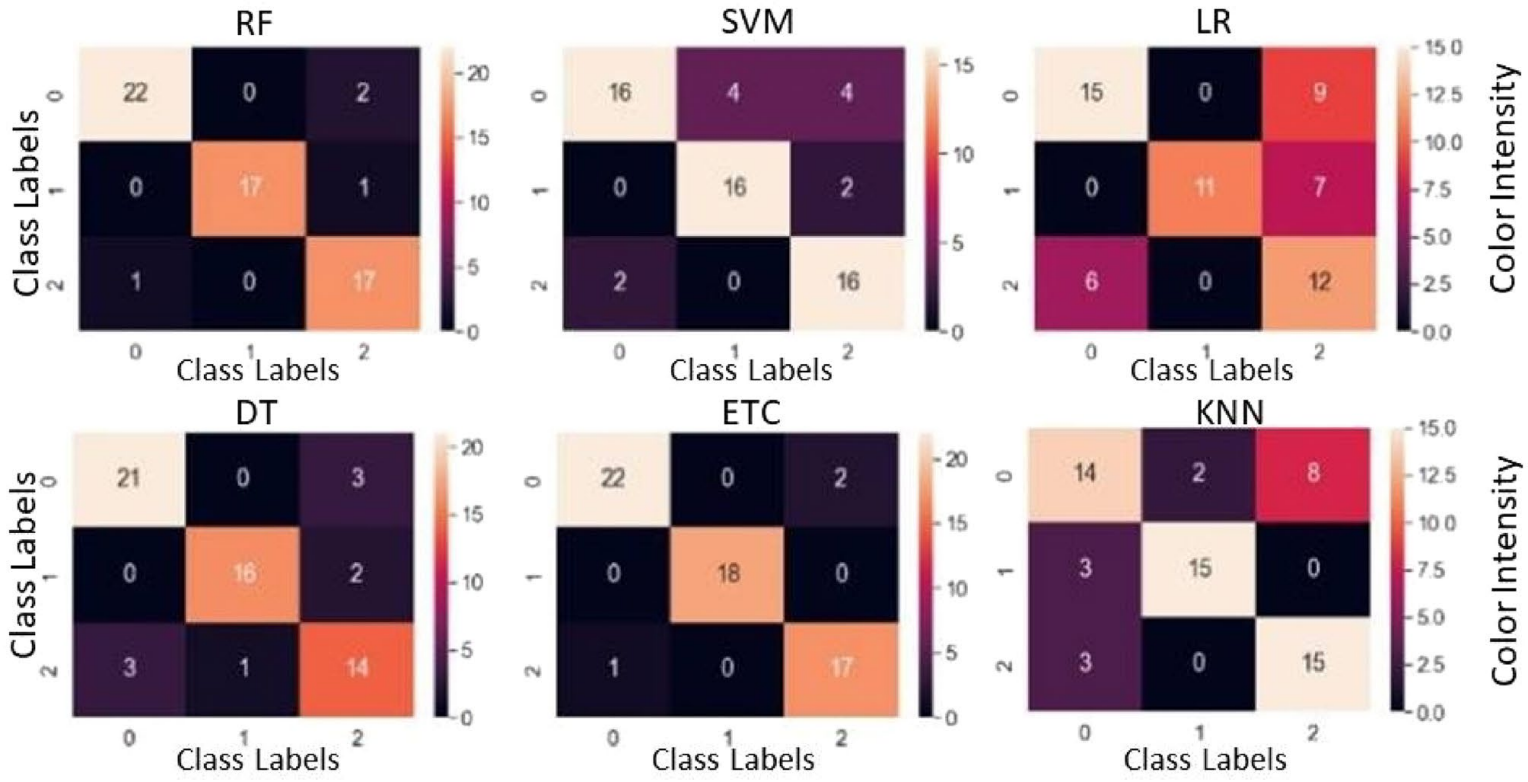
Confusion matrices for machine learning models where the “0” label represent Aas-2011, the “1” label represents Miraj-08 and the “2” label represent Punjnad-01.

The results of machine learning models are presented in Table 7; each model's confusion matrix for detailed accuracy of each class is shown in Fig. 6. According to the results, the performance of machine learning models is also good as tree-based models RF, ETC perform significantly better with 96% and 95% accuracy scores, respectively. RF, ETC are tree-based ensemble models that perform significantly even on small-size datasets. LR and SVM show poor performance because they need a large feature set for the good fit of models.

**Table 7:** Performance of machine learning models.

Model	Accuracy	Precision	Recall	F1 score
ETC	0.95	0.94	0.91	0.89
RF	0.96	0.93	0.92	0.87
SVM	0.84	0.81	0.83	0.82
DT	0.85	0.84	0.83	0.80
LR	0.63	0.71	0.63	0.65
KNN	0.73	0.72	0.78	0.75

The accuracy of the RF algorithm is the second highest in terms of accuracy with Kappa statistics. These studies^93,94^ compared their results with Kappa statistics with less than 88% satisfaction. At the same time, the results show that the samples are more consistent and reliable as compared to the above researchers. It means that MSR5 data has more potential to classify at the micro class classification level without any overlapping of various types of varieties with a minimum error rate. SVM is also a well-known classifier used in LULC classification^95,96^ with various kernels. For this research, the performance of SVM is not significant as its accuracy is 80%, which is only better than KNN and LR.

**Figure 7:**
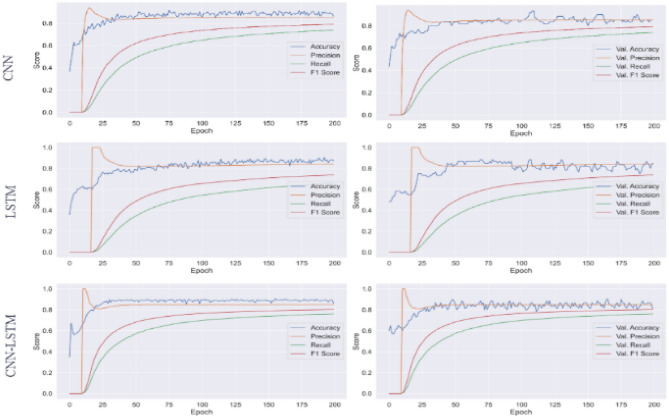
The accuracy, precision, recall, and F1 score of the deep learning models on the training data are presented in the left column, while the results on the testing data are displayed in the right column.

We deploy several deep learning models to predict wheat yield varieties, such as long short-term memory (LSTM), convolutional neural networks (CNN), and CNN-LSTM. These models are used in comparison with the proposed ANN model. Each model consists of an embedding layer with a vocabulary size of 100,000 and output dimensions of 200. After the embedding layer, the LSTM model contains a dropout layer with a 0.5 dropout rate, which will randomly remove 50% of neurons to reduce the complexity. The LSTM layer with 100 units is followed by the 100 units and in the end, the LSTM model has a dense layer with three neurons and a Softmax function. CNN model contains a 1D convolutional layer after embedding layer with 128 filters, 3 × 3 kernel size, and ReLU (rectified linear unit). A max-pooling layer with a 3 × 3 pool size is used after the 1D convolutional layer to extract the important feature set. The max-poolingThe max-pooling layer follows ReLU activation layer follows ReLU activation layer and then a dropout layer is used with a 0.5 dropout rate. A flattening layer is used to convert 3-dimensional data into a 1-dimensional layer. In the end, we use a dense layer with three neurons and a Softmax function. For CNN-LSTM, after the embedding layer we used 1D convolutional layer with a max-pooling layer and activation layer then we used the LSTM layer with 100 units. Similarly, in the end, we used a dense layer with three neurons and a Softmax function. We compile all models with Adam optimizer and 'categorical cross-'entropy' loss function. We fitted each model with 200 epochs. The accuracy, precision, recall, and F1 score of deep learning models are given in Fig. 7.

Table 8 contains the results for the deep learning models, which indicate that LSTM achieved 83% accuracy and CNN achieved 88% accuracy, which is better than LSTM. The performance of CNN-LSTM is not good as compared to individual CNN. Overall, the performance of LSTM, CNN, and CNN-LSTM is not good compared to ANN because these models require a large dataset with a large feature set.

**Table 8:** Performance of deep learning models.

Model	Accuracy	Precision	Recall	F1 Score
LSTM	0.83	0.84	0.80	0.78
CNN	0.88	0.89	0.86	0.84
CNN-LSTM	0.87	0.83	0.84	0.85

**Figure 8:**
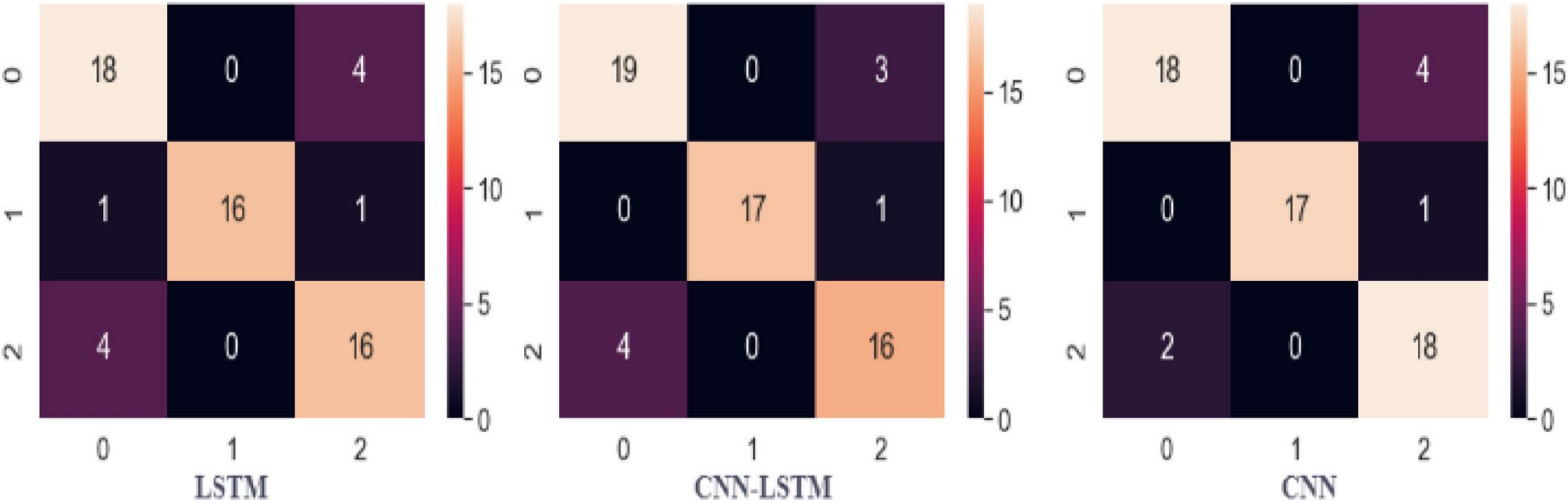
Confusion matrices for deep learning models where the “0” label represents Aas-2011, the “1” label represents Miraj-08, and the “2” label represent Punjnad-01.

Figure 8 shows the confusion matrices for wheat crop variety prediction for LSTM, CNN, and CNN-LSTM models. It can be observed that the number of highest correct predictions come from the CNN model, followed by the CNN-LSTM while the LSTM modelLSTM modelLSTM modelLSTM model gives the lowest number of correct predictions gives the lowest number of correct predictions gives the lowest number of correct predictions. On average, the performance of deep learning models is inferior to machine learning models^97–99^.

## Discussion

This case study explores the effectiveness of multispectral radiometers used in remote sensing and crop monitoring system. One of the most important advantages of MSR5 is that it has a fine spatial resolution and is easily implemented in a small study area with a controlled environment^100,101^. For conducting the pilot study, three plots of wheat crop species were cultivated, and take temporal images after fifteen days to prepare the spectral data set of wheat crop varieties used in remote sensing to find the capacity of MSR5 for micro-class classification. The results are shown in Fig. 4, three clusters of micro-class varieties of wheat crop yield data point using PCA, which indicates that all the sample points are well clustered with low variation within the class. A large distance is also found between class to class ‘Aas-'2011’, ‘Mirage-'08’, and ‘Punjnad-'1’ of wheat varieties. Figure 4 shows that five samples of ‘Aas-'2011’ numbered with labeled one are dispersed from the center of a big cluster of ‘Aas-'2011’. Due to this, the performance of models is slightly affected. Maybe these sample points are recorded with noise due to light or sensor movement during the scanning process or with tree shadow/ appearance of a cloud. However, it shows the maximum potential of MSR5 to classify the wheat crop varieties, which is the first goal of this research^60,93,94,102–104^.

The second goal of this study is to implement the various traditional machine learning models and try to find the optimal solution in terms of the accuracy and efficiency of the machine learning model achieved by implementing the ANN with various settings. Results show that we can improve the results of ANN by a small increase of the (η) rate, but results are going overfitting or underfitting. So it is proved that (η) rate change greater than 0.5 is a useless activity^58,59,65^. One to two percent accuracy can be improved by increasing the number of hidden layers that should be less or equal to the number of output classes + 1. There is no need to increase the number of first hidden layers from one to more classes to avoid the overfitting or underfitting of the model. After tuning the ANN compare its performance with a tree-based classifier and support vector machine for doing empirical analysis of various algorithms in which it is analyzed that random forest is the best model in terms of efficiency and ANN is little best in terms of accuracy^5^.

The third goal is achieved by comparing the traditional approach with the classical machine learning method results given in Tables 7 and 8, which show that ANN is better than ETC and CNN, which obtains the best results among machine learning and deep learning models. On the other hand, several researchers apply the classical method for land use land cover classification using spectral images with various indexes of spectral images like NDVI and high-resolution images^16,59,62^. It is possible that the deep learning models can performs better, with a large dataset with texture features and photographic data using data fusion techniques to improve the model’s accuracy^63^.

## Conclusions

This study demonstrates that multispectral remote sensing MSR5 can be used for micro-classifying wheat crop yield at high spatial and temporal resolution. The Statistical and agriculture related departments can utilize this study for crop mapping and trending crop varieties to get and promote high-quality varieties and increase the country’s production and food security. It is also helpful to find the effect of climate on various types of crop varieties using remote sensing with low cost and the minimum period before the time to manage the need for food. In machine-learning models, RF performs best with approximately 96% accuracy, followed by the ETC with a 95% accuracy score. The best performance is obtained using the ANN which achieves approximately 97% accuracy score. It's recommended to digital policymakers from the agriculture department can use ANN and RF to identify the different genotypes at farmer’s fields and research centers. The findings showed that multispectral data can map genotype to phenotype and classification of wheat varieties. This methodology may also be used for other crop mapping and genotype identification for accurate area estimation and yield forecasting at regional scale to ensure a better policy for food import and export at national level to ensure food security.

## Data Availability

All data generated or analysed during this study are included in this published article and data presented in this study are available on request from the first author (Mutiullah Jamil; email: mutiullahj@gmail.com) and corresponding author (Ahsan Raza; email: araza@uni-bonn.de).
